# Raising the profile of pilot and feasibility studies in relation to the development, evaluation and implementation of patient-reported outcome measures

**DOI:** 10.1186/s40814-017-0151-x

**Published:** 2017-06-17

**Authors:** Georgina Jones

**Affiliations:** Department of Psychology, School of Social Sciences, Leeds Beckett University, Leeds, LS1 3HE UK

## Abstract

This editorial introduces a new special series on the pilot and feasibility testing of patient-reported outcome measures (PROMs) in the on-line open access journal *Pilot and Feasibility Studies*. Pilot and feasibility studies are typically implemented to address issues of uncertainty before undertaking a larger definitive study such as a randomised controlled trial or large scale survey. This editorial considers the role that such pilot and feasibility testing plays in relation to the development, evaluation and implementation of PROMs. This is often an essential element of PROM research but is typically overlooked—especially within current methodological guidance, reporting space and also debate. This editorial aims to open up a dialogue about the role of pilot and feasibility testing in relation to PROMs. It highlights some of the areas in PROMs research where these types of studies have been carried out and discusses the ways in which the PROM community may be better supported and encouraged to integrate this element of the research process into their PROM-based work.

## Background

The application of social science methods in the evaluation of medical care has continued to grow in importance. In particular, there is an increasing demand for the design, development and implementation of questionnaires that can assess patients’ experiences of health and illness. These questionnaires are typically referred to as patient-reported outcome measures (PROMs) or patient-reported outcomes (PROs). They are questionnaires designed to provide a means of measuring the impact of illness, and its associated treatments, or other types of intervention, from the patient’s perspective.

Traditionally, medical care was evaluated using clinical measures of outcome, i.e. measures of mortality and other clinical diagnostic criteria which concentrated upon the physical components of health and ignored the dimensions of well-being and functioning, which could have an impact upon the health status of the patient [1]. However, in the latter half of the twentieth century, there was increasing awareness that health and illness are not purely dependent upon physical well-being. In 1954, the World Health Organization (WHO) emphasised this point in their definition of health as “a state of complete physical mental and social well-being and not merely the absence of disease or infirmity” [2]. Also, in the past, the evaluation of patients’ experiences of health and illness was primarily based upon the objective judgements of clinicians. It has been suggested that these judgements were often based upon intuition and personal experience [3]. However, research has shown that clinical and other such proxy reports made on behalf of a patient (e.g. from parents or carers) are far from objective and show variations and low levels of agreement to those of the patient [4–6]. As a result, there has been a growing demand to assess and evaluate the other dimensions of well-being which can impact upon the health of patients and to develop measurement tools in the form of questionnaires which can evaluate systematically this subjective impact on well-being beyond the traditional measures of outcome such as mortality or morbidity [7].

## Types and uses of PROMs

There are a large number of PROMs available, and they may differ in their measurement properties, content length and intended purpose. However, typically, they can be categorised as being generic, disease or condition specific. These may also have utility (preference) values estimated for the responses and therefore become preference-based measures. These are used for calculating quality-adjusted life years (QALYs), allowing for the economic value of interventions to be assessed [8]. More recently, with the increasing drive to capture data as part of routine care and find cost-effective, time efficient ways of routinely capturing the impact of treatments and illnesses from the patient’s perspective, there has been a rapid rise in ePROMs and a move away from the traditional mode of paper completions to electronic/web-based solutions. This is evident across a large and diverse range of medical disciplines [9] including pre-operative assessment [10], surgery [11] and cancer [12] to name a few.

PROMs have an important role in evaluative research as a measure of outcome. This particularly applies to the interpretation of RCT data where their use can provide additional information on the benefits of medical therapies or interventions, as an aid to clinical decision-making [13]. A second related factor is their use in quality assurance and audit [14]. Thirdly, they can assess the health care needs of populations by being used in surveys to capture information on the health needs of populations beyond the traditional mortality and socio-demographic data which are not specific enough to inform decision makers about the allocation of resources [15].

Most importantly, though, as PROMs are all concerned with providing information on the things which matter most to the patient, they can also provide valuable information to the clinician or other health care professional about patients’ progress. This can aid in the clinical management of the patient by enabling physicians to monitor patients’ progress and consequently influence decisions about treatment. Finally, an important use which is frequently overlooked is that completing a PROM also provides the opportunity for the patient to express the impact of the illness upon his/her well-being. For example, when PROMs have been administered in routine clinical practice, it has been found that patients appreciated the opportunity to report how they were feeling and to be involved with their care [16].

## The use of PROMs in pilot and feasibility studies

Thus, within medicine and the related disciplines, PROMs clearly have a number of important and useful roles and their use is becoming more widespread—particularly with the drive to incorporate these more routinely into clinical practice and medical decision-making [17–19]. For example, since 2009, the NHS has made it a requirement to collect PROM data from patients before and after surgery in four surgical conditions: hip replacement, knee replacement, varicose veins and groin hernia, with it recently reported that there were plans to extend the PROMs programme over a wider range of long-term conditions and treatments in the NHS, including mental health, chronic obstructive pulmonary disease (COPD), diabetes, epilepsy, heart failure and stroke [19].

Guidelines for conducting pilot and feasibility studies have been published by both the Medical Research Council (MRC) and National Institute for Health Research (NIHR) [20], with the MRC reporting that pilot and feasibility testing are interchangeable concepts covering all aspects of preparatory work in their guidelines on complex interventions (www.mrc.ac.uk/complexinterventionsguidance). The pilot and feasibility testing of PROMs often pla﻿ys ﻿an essential role. For example, in terms of the evaluation and implementation of the DH PROM programme the measures were only selected by the Department of Health following testing in numerous pilot studies [21] based upon a process which involved “piloting their use and reviewing their potential to be rolled out across the NHS” [19]. Similar projects have also been or are currently underway to evaluate the feasibility of implementing PROMs into current practice. For example, one initiative is “The cardiac revascularisation PROMs pilot” [22]. This was originally commissioned by the Department of Health in 2011 but later passed to NHS England in 2013. Patients across 11 English NHS hospital trusts who were on a waiting list for cardiac revascularisation to treat their heart disease were invited to participate in the PROM pilot. The aim of this was to evaluate if it was possible for the NHS to collect good enough data pre- and post treatment. A few other similar feasibility studies have been published which have particularly focused on determining the feasibility of implementing PROM/s and estimating response rates—although other important aspects such as gaining stakeholder and service user feedback, exploring responsiveness and estimating costs associated with the PROM completions were also some key aims of the pilot stage [23–25].

However, less guidance and debate exists on the ways that pilot and feasibility testing can be integrated into all aspects of PROM research. For example, although there is no single correct way to develop a PROM measure, the Food and Drug Administration (FDA) provided a figure to summarise and describe the iterative process that can be involved during PROM development [26]. Within this process, the development of PROMs involves five overarching stages where pilot and feasibility testing could play an important methodological role ((1) Hypothesize Conceptual Framework, (2) Adjust Conceptual Framework and Draft Instrument, (3) Confirm Conceptual framework and Assess other measurement properties, (4) Collect, Analyse and Interpret Data, and (5) Modify instrument). However, it is only in relation to stage 2 and establishing content validity and confirming the conceptual framework of a PROM that the FDA specifically recommends the importance of undertaking pilot studies. They recommend there should be an examination of:

all items and procedures in a pilot test of whether patients understand the items and instructions included in the PRO instrument [26].

The FDA recommends cognitive interviewing as one such way of carrying out this assessment and undertaking other small pilot studies to test the face validity of the measure (e.g. that response options and recall periods are appropriately comprehended and that the instrument’s readability is adequate for the intended population). However, more guidance on what the nature of these pilot and feasibility studies may entail is not provided. Similar gaps are evident in other key user manuals and checklists within the PROM field, e.g. COSMIN [27, 28]. It is unclear in these international guidelines how pilot and feasibility studies may be used to support the methodological quality of studies that are focused on PROM development, evaluation and implementation, or if the same recommendations as they currently stand in these documents translate to the pilot and feasibility testing of PROMs also.

Despite this, it appears as though researchers are using their own initiatives to incorporate pilot and feasibility testing. By no means an exhaustive list, some examples of these initiatives include (i) developing questionnaires to determine the relevance and acceptability of PROMs during aspects of face validity [29]; (ii) testing search strategies in systematic reviews of PROM measures and literature [30]; (iii) carrying out a pilot study to identify the domain structure of a measure and establishing the psychometric properties of the instrument (e.g. as part of demonstrating aspects of reliability, validity and responsiveness) [31], establishing other key aspects such as costs and generating stakeholder feedback [23–25]; and (iv) piloting the PROM as part of a feasibility study to inform the design of a larger definitive randomised control trial [13]. With the rise in ePROMs, there is also more demand to undertake feasibility testing to test the equivalence of administering an e-version of a PROM compared to its paper version (although the need for such testing has been questioned [32, 33]) and/or establish its acceptability and usability as part of routine clinical care [34, 35].

## Special series

Despite the essential role that pilot and feasibility testing plays in relation to PROMs, little attention to date has been given to this important stage. It would seem that there are missed opportunities to provide more guidance and ideas regarding what types of pilot or feasibility tests could be carried out. In particular, this is in relation to the ways in which pilot and feasibility tests can be integrated during PROM development, evaluation and implementation and also in terms of what the “standards” are for assessing the methodological quality of these types of studies.

This series seeks to provide a forum for the research community to share and disseminate the work they have been doing which concerns the pilot and feasibility testing of PROMs. This is often reported as one small step during the PROM reporting process and may be the result of peer review journals not considering this stage of high enough importance to dedicate publication space. Therefore, this series aims to give researchers the ability to dedicate the reporting space needed to fully report the processes undertaken, raise the profile of pilot and feasibility testing in relation to PROM research and to provide a platform on which innovative methods can be shared. By dedicating more publication space to the reporting of pilot and feasibility studies in relation to PROMs, it may also help to open up a dialogue amongst the PROM research community about some of the academic and practical issues raised above.

In the future, hopefully, the pilot and feasibility testing of PROMs can align more fully with the MRC guidance so that (i) this crucial stage of the research process can be integrated across all aspects of preparatory work related to PROM development, evaluation and implementation and (ii) clearer guidance and benchmarks for conducting such pilot and feasibility studies can become available to support the research community whilst carrying out these important stages during their PROM-based research.
